# Construction and validation of a nomogram prediction model for postoperative incisional infection in ankle fractures

**DOI:** 10.1097/MD.0000000000036408

**Published:** 2023-12-01

**Authors:** Guang-Hua Deng

**Affiliations:** a Ya’an Hospital of Traditional Chinese Medicine, Xi'an City, Shaanxi Province, China.

**Keywords:** ankle joint, fracture, infection, nomogram

## Abstract

The aim was to investigate the independent risk factors for postoperative incisional infection in ankle fractures and to establish a nomogram prediction model accordingly. Data were collected from ankle fracture patients in the Affiliated Hospital of Xinjiang Medical University from January 2018 to December 2022. Univariate and multivariate logistic regression analyses were used to determine the independent risk factors for postoperative incisional infection in ankle fractures and to establish the corresponding nomogram. Receiver operating characteristic curves were plotted and area under the curve was calculated, and calibration curves and decision curve analysis were plotted to evaluate the model performance. A total of 722 patients with ankle fractures were included in the study, and 76 patients developed postoperative incisional infections, with an incidence of 10.53%. After univariate and multivariate logistic regression analysis, a total of 5 variables were identified as independent risk factors for postoperative incisional infection in ankle fractures, namely, age ≥ 60 years (OR, 1.885; 95% CI, 1.156–3.045), having diabetes (OR1.625; 95% CI, 1.095–2.876), open fracture (OR, 5.564; 95% CI, 3.099–9.990), albumin < 35 g/L (OR, 2.618; 95% CI, 1.217–4.215), and operative time ≥ 2 hours (OR, 1.606; 95% CI, 1.077–3.247). The nomogram for postoperative incisional infection after ankle fracture constructed in this study has good predictive accuracy and helps orthopedic surgeons to intervene earlier in patients at high risk of postoperative incisional infection after ankle fracture.

## 1. Introduction

Ankle fractures are very common among all types of fractures.^[1,2]^ Most of the time, fractures are caused by violent factors such as traffic accidents and falls from heights.^[3,4]^ The common clinical symptoms are pain and swelling of the ankle joint, and sometimes deformity and dislocation of the ankle joint.^[5]^ After an ankle fracture, the patient has difficulty walking and needs to go to the hospital for timely treatment to resume a normal life. Surgery is the main and most effective treatment for ankle fractures.^[6–8]^ However, due to anatomical reasons,^[9,10]^ the soft tissues in the ankle joint are relatively weak and the blood supply is poor, which leads to a high risk of infection in the postoperative incision of the ankle joint. According to studies, the incision infection rate after ankle fractures is high.^[11]^ The occurrence of postoperative incision infection may affect the treatment outcome and increase the cost of treatment for patients. Nomograms have been used in many fields to predict not only postoperative lower limb deep vein thrombosis,^[12]^ but also postoperative pneumonia and postoperative delirium after fracture,^[13,14]^ and it has achieved good prediction results. This study aimed to investigate the independent risk factors for postoperative incisional infection after ankle fracture and to develop a nomogram prediction model accordingly.

## 2. Information and methods

### 2.1. Data sources and data collection

This study retrospectively analyzed the data of patients with inpatient surgical ankle fractures from January 2018 to December 2022 at the Affiliated Hospital of Xinjiang Medical University. Relevant information on fracture patients was collected, including patient age, gender, body mass index, smoking, alcohol, diabetes, hypertension, heart disease, open fracture, preoperative leukocytes, preoperative albumin, preoperative use of antimicrobial drugs, ASA classification, duration of surgery, and intraoperative bleeding. The study was approved by the ethical review committee of the Affiliated Hospital of Xinjiang Medical University.

### 2.2. Inclusion and exclusion criteria

Inclusion criteria: First, diagnosis of ankle fracture; second, patients with ankle fracture admitted within 72 hours after injury; third, no previous history of related surgery; 4th, postoperative incisional infection occurred by diagnostic criteria of infection; and fifth, complete preservation of patient data.

Exclusion criteria: First, the choice of nonsurgical treatment; second, multiple fractures; and third, other acute or chronic infectious diseases or severe organ damage.

### 2.3. Statistical analysis

The collected data were randomly divided into a training set (70%) and a validation set (30%) according to the ratio of 7:3 in R (4.2.1) software. The differences between infected and uninfected groups were analyzed univariately in the training set using SPSS 26.0 software, and the chi-square test was used to statistically analyze the count data. Variables screened at *P* < .05 from the univariate analysis were included in the multivariate logistic regression analysis. Variables with *P* < .05 in the multivariate logistic regression analysis identified independent risk factors for ankle fracture surgery incision infection. The screened independent risk factors were plotted in the nomogram in R software, receiver operating characteristic curves were plotted and the area under the curve was calculated in the training and validation sets, and calibration curves and decision curves analysis were plotted to assess model performance.

## 3. Results

### 3.1. General information

A total of 722 ankle fracture patients were included in this study, and a total of 76 fracture patients had postoperative incisional infections, with a postoperative incisional infection rate of 10.53%. According to the ratio of 7:3, 506 and 216 fracture patients were randomly divided into modeling and validation groups.

### 3.2. Independent risk factors for incisional infection in ankle fracture surgery

In the training set, 15 variables were analyzed by univariate logistic regression analysis, and the results showed that 10 variables were potential risk factors for incisional infection, including age, body mass index, smoking, diabetes, hypertension, open fracture, preoperative albumin, preoperative use of antimicrobials, ASA classification, and duration of surgery (Table 1). Multivariate logistic regression analysis determined that age ≥ 60 years (OR, 1.885; 95% CI, 1.156–3.045), having diabetes (OR, 1.625; 95% CI, 1.095–2.876), open fracture (OR, 5.564; 95% CI, 3.099–9.990), albumin < 35 g/ L (OR, 2.618; 95% CI, 1.217–4.215), and operative time ≥ 2 h (OR, 1.606; 95% CI, 1.077–3.247) were independent risk factors for postoperative incisional infection in ankle fractures (Table 2).

**Table 1:** Univariate analysis of postoperative incisional infection in ankle fractures.

Risk factor	Infection set (53)	Uninfected set (453)	*P* value
Age [n (%)]			<.001
<60 yr	25 (47.17%)	324 (71.52%)	
>=60 yr	28 (52.83%)	129 ((28.48%)	
Gender [n (%)]			.930
Male	24 (45.28%))	208 (45.92%)	
Female	29 (54.72%)	245 (54.08%)	
BMI [n (%)]			<.001
<24	20 (37.74%)	298 (65.78%)	
>=24	33 (62.26%)	155 (34.22%)	
Smoking [n (%)]			.032
No	36 (67.92%)	365 (80.57%)	
Yes	17 (32.08%)	88 (19.43%)	
Alcohol [n (%)]			.569
No	35 (66.04%)	281 (62.03%)	
Yes	18 (33.96%)	172 (37.97%)	
Diabetes [n (%)]			<.001
No	32 (60.38%)	367 (81.02%)	
Yes	21 ((39.62%)	86 (18.98%)	
Hypertension [n (%)]			.045
No	33 (62.26%)	340 (75.06%)	
Yes	20 (37.74%)	113 (24.94%)	
Heart disease [n (%)]			.697
No	45 (84.91%)	375 (82.78%)	
Yes	8 (15.09%)	78 (17.22%)	
Open fracture [n (%)]			<.001
No	40 (75.47%)	416 (91.83%)	
Yes	13 (24.53%)	37 (8.17%)	
Preoperative leukocytes [n (%)]			.508
<10 × 10^9^/L	31 (58.49%)	286 (63.13%)	
>=10 × 10^9^/L	22 (41.51%)	167 (36.87%)	
Preoperative albumin [n ((%)]			.004
>=35g/L	35 (66.04%)	374 (82.56%)	
<35g/L	18 (33.96%)	79 (17.44%)	
Antimicrobial drugs [n (%)]			.017
Yes	10 (18.87%)	39 (88.61%)	
No	43 (81.13%)	414 (91.39%)	
ASA分级≥3 [n (%)]			<.001
No	29 (54.72%)	377 (83.22%)	
Yes	24 (45.28%)	76 (16.78%)	
Duration of surgery [n (%)]			.003
<2 h	22 (41.51%)	285 (62.91%)	
>=2 h	31 (58.49%)	168 (37.09%)	
Intraoperative bleeding [n (%)]			.257
<100 mL	33 (62.26%)	245 (54.08%)	
>=100 mL	20 (37.74%)	208 (45.92%)	

BMI = body mass index.

**Table 2:** Multifactorial analysis of postoperative incisional infection in ankle fractures.

Risk factor	HR	CI 95%	*P* value
Age			
<60 yr	Reference	-	-
>=60 yr	1.885	1.156–3.045	.021
BMI			
<24	Reference	-	-
>=24	3.007	0.697–12.962	.140
Smoking			
No	Reference	-	-
Yes	2.246	0.267–18.920	.457
Diabetes			
No	Reference	-	-
Yes	1.625	1.095–2.876	.042
Hypertension			
No	Reference	-	-
Yes	1.112	0.510–2.425	.789
Open fracture			
No	Reference	-	-
Yes	5.564	3.099–9.990	<.001
Albumin			
>=35g/L	Reference	-	-
<35g/L	2.618	1.217–4.215	.007
Antimicrobial drugs			
Yes	Reference	-	-
No	1.965	0.935–4.132	.075
ASA classification			
No	Reference	-	-
Yes	1.478	0.565–3.866	.425
Operation time			
No	Reference	-	-
Yes	1.606	1.077–3.247	.033

BMI = body mass index.

### 3.3. Nomogram development and validation

**Figure 1.**
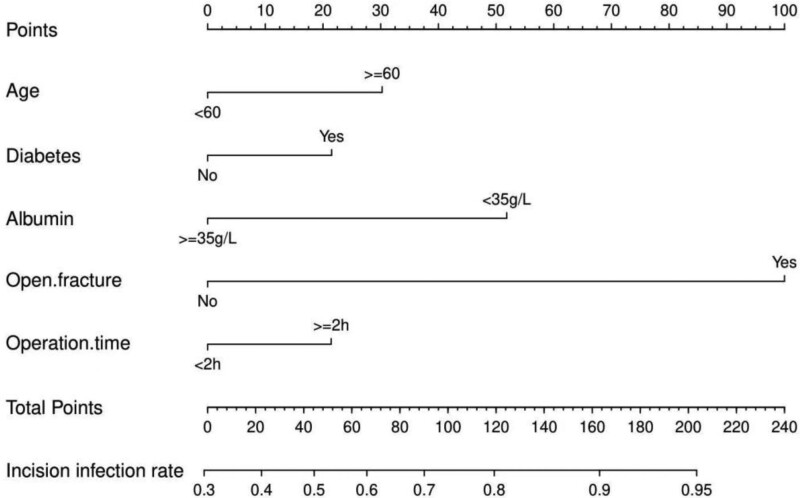
Nomogram for predicting the risk of incisional infection after ankle fracture surgery.

**Figure 2.**
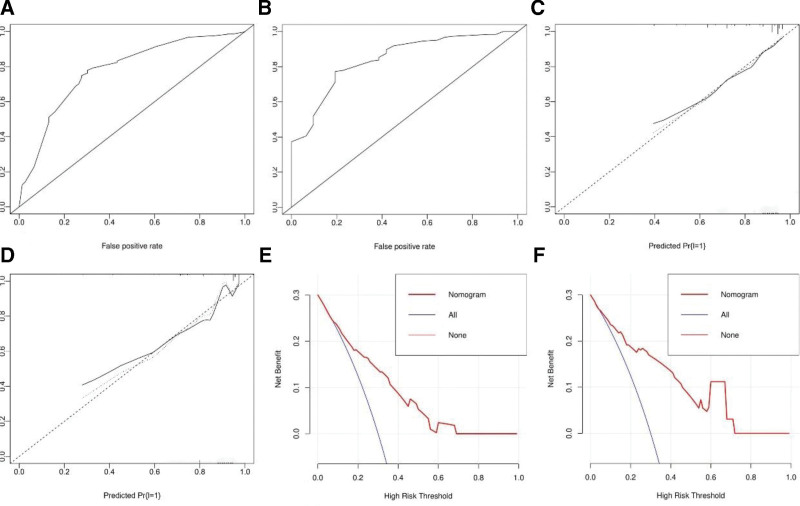
ROC curves of the nomogram for predicting incisional infection in the training set (A) and the validation set (B). Calibration curves of the nomogram for predicting incisional infection in the training set (C) and the validation set (D). DCA of the Nomogram for predicting incisional infection in the training set (E) and the validation set (F). DCA = decision curve analysis, ROC = receiver operating characteristic.

A nomogram was drawn using screened independent risk factors and used to predict the risk of postoperative incisional infection in ankle fractures (Fig. 1). Receiver operating characteristic curves were then plotted for the training and validation sets, and the corresponding area under the curves were calculated to be 0.778 and 0.842 (Figure 2A, B). In addition, the calibration curves were plotted, indicating that the nomogram-predicted risk agreed well with the actual risk of occurrence and had good predictive ability (Figure 2C, D). Also, decision curve analysis showed that the nomogram had a good predictive ability (Figure 2E, F).

## 4. Discussion

Despite current advances in operating room aseptic conditions, sterilization methods, protective measures, surgical techniques, and routine antibiotic prophylaxis protocols, incisional infections remain the most common and costly postoperative fracture complication.^[15,16]^Incisional infections lead to an increased risk of internal fixation infection when the internal fixation needs to be removed, which can increase complications and mortality, as well as the economic and social burden. The current study collected clinical data from patients with ankle fractures over the past 5 years to develop a clinical prediction model for predicting the risk of incisional infection after ankle fracture surgery.

In this study, we used predictors that are common and easily identified in clinical practice, and developed this nomogram prediction model based on 5 independent risk factors identified, and model validation determined that the model developed in this study has good predictive power.

The results of the study showed that age is a risk factor for postoperative incisional infection in patients with ankle fractures. The body’s functions decline with age, the body’s immunity and tissue repair capacity decrease, and the body’s resistance decreases.^[17]^ The combined underlying disease is also a susceptible cause of postoperative incisional infections.^[18]^

The results of the study showed that having diabetes is a risk factor for postoperative incisional infection in patients with ankle fractures. Patients with diabetes often have decreased body immunity and poor body composition, and elevated blood glucose provides a good environment for bacteria to survive and reproduce.^[19]^ At the same time, diabetes can cause vascular lesions in the body, especially in the distal extremities, which are more prone to incisional infections. Studies have confirmed that the risk of postoperative infection increases by 10% to 50% in patients with diabetes mellitus.^[20]^ Therefore, blood glucose needs to be controlled within the ideal range during the surgical period to reduce the risk of incisional infection.

The results of the study showed that open fractures are a risk factor for postoperative incisional infections in patients with ankle fractures. There is a potential risk of skin or mucous membrane rupture at the open fracture, and the fracture end is connected to the outside world, which makes it easy for bacteria to enter the local tissues.^[21]^ When the body is weak or the local blood transport is poor, the latent bacteria will multiply rapidly and lead to incisional infection.^[22]^ Therefore, open fractures should be debrided as early as possible, and those with more serious injuries should be debrided in the operating room, while removing soft tissues that have lost blood flow and removing foreign bodies.

The results of the study showed that decreased preoperative albumin is a risk factor for postoperative incisional infection in patients with ankle fractures. Albumin is an important nutrient for the body, maintaining the patient’s plasma colloid osmotic pressure, regulating cellular metabolism, and ensuring normal immune function. Its low level will lead to a decrease in the patient’s immune capacity and increase the risk of infection. Postoperative patients with ankle fractures have increased nutrient requirements and decreased wound healing ability, making them more prone to incisional infections.^[23,24]^ It is recommended to do preoperative testing of albumin indexes and to prepare a response plan in advance, such as giving nutritional preparations for intensive nutritional support after surgery to improve patients’ immunity and reduce the risk of postoperative incisional infection.

The results of the study showed that prolonged operative time was a risk factor for postoperative incision infection in patients with ankle fractures. It has been shown that the longer the surgery, the greater the probability of postoperative incisional infection in patients.^[25]^ The long operative time indicates the difficulty of surgery, the need for intraoperative dissection of the operative area, and the increased degree of damage to soft tissues. Exposure the incision for a long time increases the risk of infection. To address this risk factor, it is important to adequately prepare for surgery, strictly enforce intraoperative aseptic operation, reduce bleeding, minimize the operative time, and perform staged surgery if necessary to reduce the incisional infection.

However, there are shortcomings in this study. First, because this is a retrospective study, there will be some unavoidable errors arising. Second, this was a single-center study conducted at a tertiary referral trauma center, and there was bias in the selection of patients due to more severe fractures or more complex conditions in the admitted patients. Third, this is a risk prediction model developed in a single-center, and therefore its validity needs to be validated in further multicenter studies.

## 5. Conclusion

The nomogram for postoperative incisional infection after ankle fracture constructed in this study has good predictive accuracy and can help orthopedic surgeons to intervene in advance for patients at high risk of postoperative incisional infection after ankle fracture.

## Author contributions

**Conceptualization:** Guang-Hua Deng.

**Data curation:** Guang-Hua Deng.

**Formal analysis:** Guang-Hua Deng.

**Funding acquisition:** Guang-Hua Deng.

**Investigation:** Guang-Hua Deng.

**Methodology:** Guang-Hua Deng.

**Project administration:** Guang-Hua Deng.

**Resources:** Guang-Hua Deng.

**Software:** Guang-Hua Deng.

**Supervision:** Guang-Hua Deng.

**Validation:** Guang-Hua Deng.

**Visualization:** Guang-Hua Deng.

**Writing – original draft:** Guang-Hua Deng.

**Writing – review & editing:** Guang-Hua Deng.
